# Neglected isolated plantar dislocation of middle cuneiform : a case report

**DOI:** 10.1186/1471-2474-8-5

**Published:** 2007-01-17

**Authors:** Ashu Verma, Vinod Kumar Sharma, Sumit Batra, Mahender Singh Rohria

**Affiliations:** 1 Department of Orthopaedic Surgery, Central Institute of Orthopaedics, Vardhaman Mahavir Medical College and associated Safdarjung Hospital, New Delhi-29, India

## Abstract

**Background:**

Four cases of plantar dislocation of middle cuneiform have been reported in the english literature. All of them were fresh cases and treated with open reduction. We are reporting a case of neglected plantar dislocation of middle cuneiform which was treated with excision.

**Case presentation:**

A farmer presented with a painful plantar dislocation of middle cuneiform bone after 9 months of injury. The bone was deformed and was excised by a plantar incision. It resulted in painless foot with no disability.

**Conclusion:**

The neglected plantar dislocated middle cuneiform bone becomes deformed due to repeated weight bearing. The gap gets filled with Fibrous tissue. Excision of the cuneiform gives good results.

## Background

Middle cuneiform can dislocate plantarwards by a strong vertical force which flattens the arches of foot and also breaks the ligamentous support as has been reported in the literature [1]. Four cases of plantar dislocation of middle cuneiform have been reported in the english literature that presented as fresh cases [1-4]. All of them were treated with open reduction. We are presenting a case of neglected plantar dislocation of middle cuneiform bone.

## Case presentation

**Figure 1:**
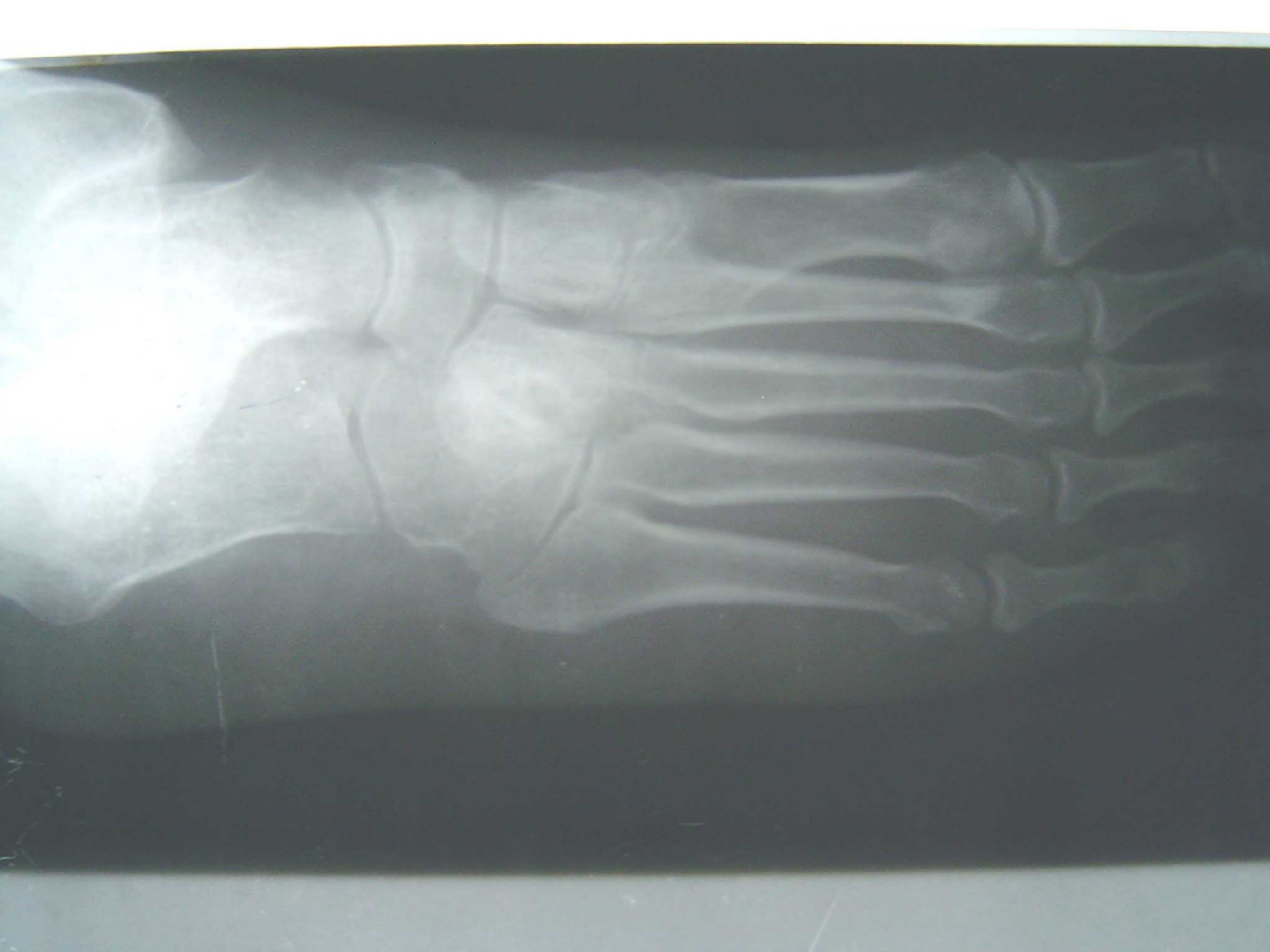
AP view of foot showing empty space in the region of middle cuneiform.

**Figure 2:**
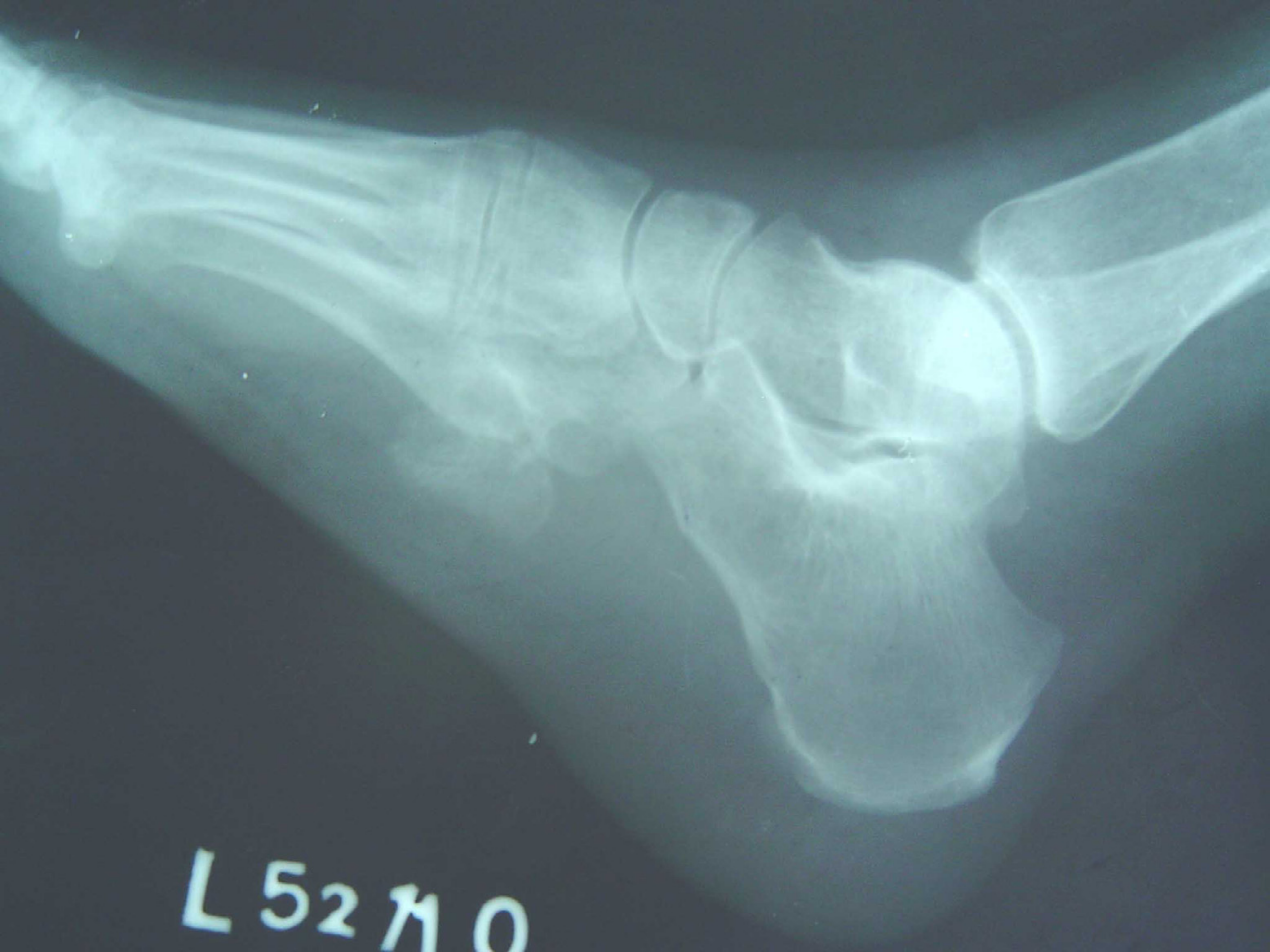
Lat view of the foot showing plantar dislocation of middle cuneiform.

A 48 year old farmer was hammered with an iron rod over the dorsum of foot 9 months back. He presented with a painful swelling on the plantar aspect of left foot inferolateral to the medial cuneiform. It was his first presentation to a qualified medical practitioner since the time of injury. The swelling was bony hard in the subfascial plain and mobile in all directions. It was tender on palpation. The overlying skin was thin and stretched. Radiographs showed plantar dislocation of middle cuneiform (figure 1 &2). The other bones of foot were well aligned and stable.

A plantar longitudinal incision was made over the swelling. An inflamed bursa was excised and the bone was dissected out. It was deformed and irregular. The space between medial and lateral cuneiform was filled with fibrous tissue. The relations between rest of the bones were maintained and stable. The bone was excised and wound was closed. Patient had full range of motion postoperatively. Weight bearing was restricted for two weeks. After the removal of stitches full weight bearing was allowed. The patient was asymptomatic at 6 months follow up.

## Conclusion

The middle cuneiform is a wedge shaped bone. Very strong deep planar ligaments, superadded with the tendinous slips of tibialis posterior muscle and the shape of the bone with a base dorsally are the factors which provide plantar stability to the bone. Besides these the interosseous ligaments between the adjacent bones and the dorsal transverse ligaments provide added stability. It receives its vascularity from dorsal surface mainly and also from medial and lateral surfaces [5].

Dislocation of middle cuneiform has been reported in middle age group males [1-4]. The mechanism for the planter dislocation is a direct vertical force to the dorsum of midfoot that compresses and flattens the transverse and longitudinal arch. The resultant force may concentrate at intermediate cuneiform and ligament injury may be limited to the ligaments around the intermediate cuneiform [1]. The plantar dislocation of bone detaches the dorsal vessels supplying the bone and leads to avascular necrosis.

The dislocated bone gets deformed due to repeated weight bearing. Deformed bone, mechanical impingement of the bone and bursa formation around the bone are the factors causing pain.

The bone was deformed in our case and ligamentous healing with fibrosis was noticed on surgical exploration. It was decided not to attempt open reduction in this case because of high possibility of avascular necrosis if the dislocated bone was reduced. Also arthrodesis between tarsal bones has been associated with poor results [6]. Hence a decision was made to excise the dislocated bone which showed good result. The patient was seen at six months after surgery and was aymptomatic but long term follow up is necessary to look for any malalignment of the Lisfranc joints.

## Competing interests

The author(s) declare that they have no competing interests.

## Authors' contributions

AV conducted the surgery, collected data and has written the case report.

VKS planned and guided the surgery.

SB was involved in the planning of surgery and helped in writing the case report.

MSR assisted the surgery and compiled the data.

All authors read and approved the final manuscript.

## Pre-publication history

The pre-publication history for this paper can be accessed here:
